# Macrophages in inflammatory skin diseases and skin tumors

**DOI:** 10.3389/fimmu.2024.1430825

**Published:** 2024-12-05

**Authors:** Si-Han Liu, Jie Zhang, Ya-Gang Zuo

**Affiliations:** Department of Dermatology, State Key Laboratory of Complex Severe and Rare Diseases, National Clinical Research Center for Dermatologic and Immunologic Diseases, Peking Union Medical College Hospital, Chinese Academy of Medical Sciences and Peking Union Medical College, Beijing, China

**Keywords:** macrophage, inflammatory skin diseases, skin tumors, pathogenesis, treatment

## Abstract

Macrophages, as specialized, long-lasting phagocytic cells of the innate immune system, have garnered increasing attention due to their wide distribution and various functions. The skin, being the largest immune organ in the human body, presents an intriguing landscape for macrophage research, particularly regarding their roles in inflammatory skin diseases and skin tumors. In this review, we compile the latest research on macrophages in conditions such as atopic dermatitis, psoriasis, systemic sclerosis, systemic lupus erythematosus, rosacea, bullous pemphigoid, melanoma and cutaneous T-cell lymphoma. We aim to contribute to illustrating the pathogenesis and potential new therapies for inflammatory skin diseases and skin tumors from the perspective of macrophages.

## 1 Introduction

Macrophages are present in all tissues of adult animals (1). They have crucial roles in an organism’s biology, including development, maintaining homeostasis, facilitating repair, and reacting to immunological assaults from pathogens. M0 macrophages, as the unmature and inactive form, polarize in different directions depending on the surrounding microenvironment, and form distinguished macrophage subtypes, such as M1 and M2 phenotype (2).

M1 macrophages, also known as classically activated macrophages, can be polarized by lipopolysaccharide (LPS) either alone or in synergism with interferon (IFN)-γ. M1 macrophages are characterized by an enhanced capacity to secrete pro-inflammatory cytokines such as interleukin (IL)-1β, IL-6, and IL-12. Phenotypically, M1 macrophages exhibit significant levels of cluster of differentiation (CD)68, CD80 and CD86. M1 macrophages play an essential role in promoting inflammation, and display anti-infection and anti-tumoral activity. However, they can also mediate reactive oxygen species (ROS)-triggered tissue impairment, affecting tissue regeneration and wound recovery.

M2 macrophages, also known as alternatively activated macrophages, are polarized by IL-4 and IL-13. They display an anti-inflammatory cytokine profile with elevated levels of IL-10 and transforming growth factor (TGF)-β. Based on the stimuli, M2 macrophages can be categorized into four subgroups, and they vary in terms of surface markers, released molecules, and biological roles. However, it is important to note that all M2 macrophages share the characteristic of co-express IL-10. M2 macrophages are crucial for clearing parasites, modifying tissues, promoting angiogenesis, and contributing to allergy disorders (3, 4).

Inflammatory skin diseases are a group of diseases resulting from immune system disorders and cause damage to skin tissue, including atopic dermatitis, psoriasis, systemic sclerosis, systemic lupus erythematosus, rosacea, bullous pemphigoid. Macrophages are recognized as significant cellular contributors to persistent inflammation across diverse tissues and illnesses (5). Concurrently, skin tumors, comprising both benign and malignant neoplasms, develop from the skin simultaneously. Nevus and hemangiomas are the most common benign skin tumors, and they are not life-threatening but impact aesthetics. Skin malignancies, including malignant melanoma, basal cell carcinoma, and cutaneous T-cell lymphoma, can be deadly and demand urgent attention. The function of macrophages in the tumor microenvironment (TME) has been extensively researched in many types of tumors, including skin malignancies. Macrophages are crucial in controlling the body’s immunological response and metabolism, perhaps contributing to the development of many diseases (4, 6). This review seeks to outline recent discoveries about the role of macrophages in different inflammatory skin diseases and skin tumors.

## 2 Atopic dermatitis

The symptoms of atopic dermatitis (AD), a chronic inflammatory skin condition, include intense itching and recurrent superficial and spongiotic inflammation (7). A complicated interplay between genetic and environmental variables, including immunological response, skin barrier failure, and pruritus, may be instrumental in the pathogenesis of AD (8). Numerous investigations have revealed a robust correlation between AD and macrophages.

### 2.1 The characteristic of macrophage in AD

Using molecular imaging approaches, 2,4-dinitrofluorobenzene (DNCB) induced AD-like skin lesions have been observed to exhibit infiltrated-macrophage profile (9). The difference in macrophage polarization between skin samples from AD and psoriasis is evident. M2 macrophages were almost exclusively detected in AD samples. While traditionally regarded as an anti-inflammatory phenotype, recent study suggested that M2 macrophages contribute to the pathogenesis of AD through the secretion of CCL18, thus promoting the continued recruitment of Th2 cells and maintaining inflammation (10).

AD macrophages have lower toll-like receptor (TLR)-2 expression and less release of pro-inflammatory cytokines in response to TLR-2 ligand stimulation when compared to healthy controls. This may be a factor in AD patients’ increased vulnerability to *Staphylococcus aureus* skin infections (11). Notably, psoriasis patients also exhibit colonization with *Staphylococcus aureus*. When exposed to *Staphylococcus aureus α-toxin*, macrophages from AD patients generated less C-X-C Motif Chemokine Ligand (CXCL)10 than those from psoriasis patients. Decreased secretion of CXCL10 results in reduced Th1 polarization (12).

A distinct cluster of macrophages expressing C-C Motif Chemokine Ligand (CCL)13 and CCL18 was discovered with single-cell RNA-sequencing in the leukocyte-infiltrated region of the lesional skin in AD. Analysis of ligand-receptor interactions revealed interactions between T cells, dendritic cell (DC)s, fibroblasts, and M2 macrophages that expressed CCL13 and CCL18. This provides a thorough understanding of the immunological milieu in AD (13).

### 2.2 The pathogenic roles of the macrophages in AD

Macrophages contribute to the development of AD through a variety of processes. An important factor in human AD is CLDN1, a component of epidermal tight junctions. The association between human AD patients’ CLDN1 levels and macrophage recruitment has been elucidated by recent research. Mice with reduced CLDN1 expression levels displayed AD-like morphological traits and attracted more macrophages to the skin lesion (14). YKL-40 is a crucial inflammatory marker in type II inflammation. Compared to normal persons, AD patients’ skin had a greater level of YKL-40. Subsequent research indicated that the primary source of YKL-40 was dermal macrophages, indicating that macrophages may be involved in the pathophysiology of AD (15).

Macrophages participate in the mechanism of AD itch as well. IL-31 is a type II cytokine linked to pruritus in many dermatologic diseases. For instance, it has been reported that CD206+ M2-like macrophages are the primary producers of IL-31 in recessive dystrophic epidermolysis bullosa (16). M2 macrophages are dominant sources of IL-31 in AD as well. Moreover, AD itch is caused by a sophisticated network of periostin, basophils, thymic stromal lymphopoietin, and IL-31-expressing macrophages (17).

Autophagy of macrophages is essential for immunological regulation and has been linked to the onset of AD. Compared to wild-type mice, autophagy-related gene 5 cKO mice display deficient autophagy activity, lower cutaneous inflammation and decreased M2 macrophage infiltration. Mechanistically, deficiency of autophagy causes CCAAT enhancer binding protein beta to accumulate, which in turn stimulates the production of suppressor of cytokine signaling 1/3, ultimately suppresses the expression of the M2 marker (18).

One hallmark of AD is inflammation-mediated lymphangiogenesis, which is intimately related to macrophage recruitment. Strong macrophage chemoattractant monocyte chemoattractant protein-1 is expressed at high levels by IL-4-stimulated keratinocyte cells. Furthermore, a notable rise in dermal macrophages expressing vascular endothelial growth factor-C, a pro-lymphangiogenic factor, is observed in the AD mice model (19).

**Figure 1:**
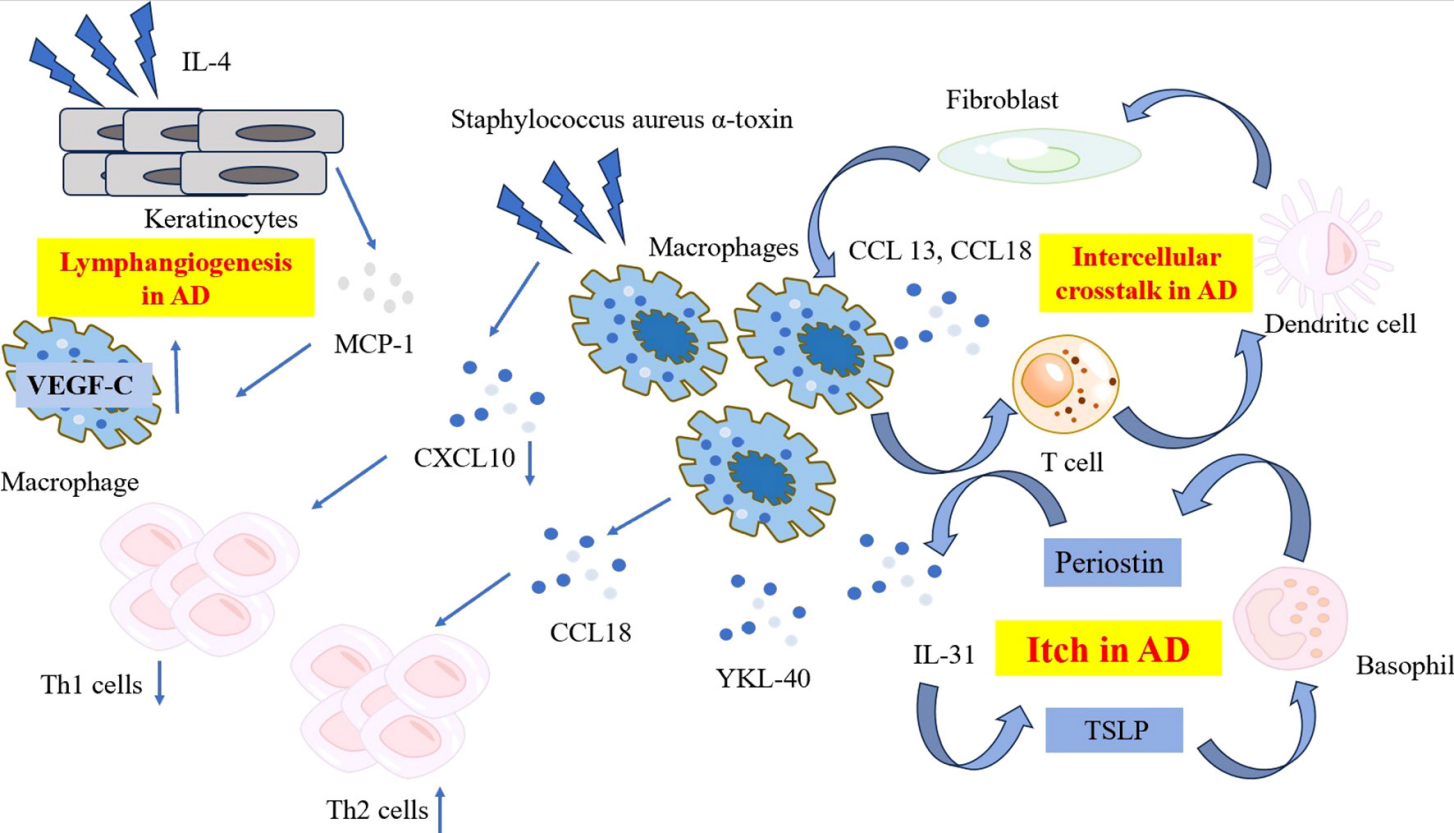
The characteristic and pathogenetic roles of the macrophages in AD. Compared to psoriasis, macrophages in AD produce lower level of CXCL10 when exposed to Staphylococcus aureus α-toxin, resulting in reduced Th1 polarization. Instead, macrophages in AD produce high levels of CCL18, recruiting more Th2 cells to affected skin and release YKL-40, an important Th2 marker. A network comprising periostin, TSLP, basophils and macrophage-derived IL-31 contribute to the mechanism of itch in AD. Ligand-receptor interactions data revealed the intracellular crosstalk between CCL13, CCL18-macrohages, T cells, DCs and fibroblasts. Macrophages also get involved in the lymphangiogenesis in AD by expressing significant level of VEGF-C. AD, atopic dermatitis; CCL, C-C Motif Chemokine Ligand; CXCL, C-X-C Motif Chemokine Ligand; DC, dendritic cell; IL, interleukin; MCP-1, monocyte chemoattractant protein-1; TSLP, thymic stromal lymphopoietin; VEGF-C, vascular endothelial growth factor-C; YKL-40, Chitinase 3-like 1.

Research has also been conducted regarding the role of chemokines related to macrophages in the etiology of AD, particularly macrophage migration inhibitory factor (MIF). The stratum corneum MIF levels in the skin lesions were found substantially higher compared to unaffected regions in the same patient. MIF provides a helpful gauge to measure the degree of AD locally (20). There is a link between the MIF promoter 173G/C polymorphism and a higher risk of AD (21). MIF promoter polymorphisms, namely the C-173 allele and the C/5-CATT and C/7-CATT haplotypes, were found to be substantially linked to a higher risk of AD in Korean patients (22). The characteristics and pathogenetic roles of the macrophages in AD are summarized in **Figure 1**.

### 2.3 Treatments for AD involving macrophages

Traditional Chinese medicine exhibited great potential in treating AD, including Periploca forrestii Schltr saponin and Stellariae Radix. Periploca forrestii Schltr saponin, which was traditionally used to treat rheumatoid arthritis, exhibits substantial potential for therapy in AD by suppressing the expression of both M1 and M2 macrophage markers (23). Stellariae Radix, which was previously used to treat fever and insomnia, successfully inhibited M1 macrophage infiltration in a DNCB-induced AD mouse model. Mechanistically, Stellariae Radix suppressed the production of tumor necrosis factor (TNF)-α, CXC-10, IL-12, and IL-1β and reduced the expression of NOD-like receptor thermal protein domain associated protein 3 (NLRP3) in M1 macrophages (24). A new topical medication for AD, called Nuclear Transport Checkpoint Inhibitor, inhibited the invasion of macrophages, and decreased the proliferation of Ki-67-positive cells (a subset of cells within the basal layer of the epidermis) (25). Naringenin, a flavonoid derived from plants, can reduce AD symptoms by inhibiting the M1-like macrophage phenotype, high mobility group box-1 (HMGB1) cascade, and levels of inflammatory cytokines. Moreover, naringenin can induce anti-inflammatory gene expression through the transformation of the M1 to M2 phenotype, resulting in increased levels of CD36 and IL-10 (26). Dictamnine, a natural alkaloid isolated from the root of Dictamnus albus, hinders DNCB-triggered AD skin inflammation by blocking M1 macrophage differentiation and enhancing macrophage autophagy at inflammation sites. Furthermore, dictamnine decreases the secretion and suppresses the genetic expression of inflammatory molecules (27). However, the curative effect of these potential therapies was evaluated in AD-mouse models and bone marrow-derived macrophages. In the future, we anticipate more large-scale clinical trials to verify these outcomes.

Nemolizumab, a humanized monoclonal antibody against IL-31 receptor A, holds great promise for alleviating pruritus and inflammation in AD patients in many clinical trials (28, 29). Dupilumab is another humanized monoclonal antibody that has gained approval for the treatment treating moderate-to-severe AD. Dupilumab can specifically bind to the IL-4Rα subunit, thereby inhibiting the signal transduction of IL-4 and IL-13, and blocking the Th2 inflammatory response. Both IL-4/13 and IL-31 pathway contributes to AD itch. Recent findings suggest that IL-31 can induce itching independently of IL-4 and IL-13 *in vivo* (30). M2 macrophages are implicated in the pathogenesis of AD pruritus and inflammation through the secretion of IL-31 and Th2 cytokines. However, there is a lack of direct studies addressing the impact of nemolizumab and dupilumab on immune cells, particularly the phenotype and number of macrophages. The janus kinase (JAK) pathway is activated in the signaling transduction of many cytokines relevant to AD. A network meta-analysis has demonstrated that many JAK inhibitors can ameliorate the signs and symptoms of AD, with upadacitinib showing particular efficacy (31). It has been documented that JAK inhibitor can reduce the infiltration of macrophages in lesional sites in allergic contact dermatitis mouse models (32). However, no analogous experiments have been conducted in AD mouse models.

## 3 Psoriasis

Psoriasis is a prevalent chronic inflammatory skin disorder distinguished by a significant inflammatory presence along with enlarged and distorted blood vessels. Infiltrated macrophages in psoriatic skin lesions are crucial in the advancement of this unregulated skin inflammation.

### 3.1 The characteristic of macrophage in psoriasis

Analyzed data from the GEO database showed a notable rise in the level of expression of macrophage markers and inflammatory cytokines in lesional tissues as compared to normal tissues in 58 patients with psoriasis (33). Significant variations in the composition of innate immune cells were found between psoriatic plaques and normal skin. There is a notable increase in the quantity of M0 and M1 macrophages in psoriatic skin. Both the count and proportion of macrophages underwent alterations. The abundance of M0 macrophages was linked to the psoriasis severity degree (34, 35). Psoriatic patients had a greater ratio of M1 to M2a macrophage polarization compared to controls (36). The proportion of C-C Motif Chemokine Receptor (CCR) 1+ macrophages increase in psoriasis-affected skin compared to healthy skin, as determined by single-cell RNA sequencing and flow cytometry data. CCR1+ macrophages exhibited elevated expression of genes associated with inflammatory cytokines and chemokines, such as CXCL-8, CXCL-2, and IL-1B (37).

Immune cell composition varies between the early and late stages of psoriatic skin lesions. Neutrophils infiltrated the epidermis in the early phase, but monocytes and monocyte-derived DCs were mostly present in the dermis. During the late phase, there was a temporary rise in the number of macrophages in the dermis (38).

### 3.2 The pathogenic roles of the macrophages in psoriasis

Several efforts have been undertaken to determine the function of macrophages in the development of psoriasis. The IL-23/IL-17 immunological axis plays an important role in the initiation and progression of psoriasis. A novel pathogenic macrophage subpopulation, triggered by IL-23 and characterized by a unique gene expression profile, has been discovered recently. M (IL-23) produce significant quantities of IL-17A, IL-22, and IFN-γ, contributing to the development of psoriasis-like dermatitis in a mouse model (39). Additionally, the IL-23/IL-17 immunological axis is proposed to play a role in the development of psoriasis by initiating ACT1/TRAF6/TAK1/NF-κB pathway in macrophages (40). Two important autoantigens in psoriasis are LL-37 and ADAMTS-Like Protein 5. It has been observed that ADAMTS-Like Protein 5+ and LL-37+ cells are co-expressed with CD163+ macrophages in both the superficial and deep dermis (41).

Interactions between macrophages and keratinocytes play a significant role in the development of psoriasis. Keratinocytes can interact with macrophages via HMGB1, promoting macrophage inflammatory polarization (42). The interaction between macrophages and exosomes generated from vitamin D receptor-deficient keratinocytes is crucial for the advancement of psoriasis. Exosomes-sh vitamin D receptor markedly enhanced macrophage proliferation and directed their polarization toward the M1 phenotype, while suppressing macrophage apoptosis (43).

Psoriasis is more prevalent and severe in men than in women. A recent investigation has shown that the root cause is linked to estrogen. Estradiol can inhibit the production of IL-1β by macrophages, and IL-1β is necessary for the generation of IL-17A in the psoriasis model. This perspective may clarify the disparity in both the occurrence and seriousness of psoriasis between genders (44).

Macrophages and psoriasis-related comorbidities have also been studied. Psoriasis patients with comorbidities have elevated levels of chitotriosidase compared to those without comorbidities. Chitotriosidase is primarily produced by activated macrophages in reaction to pro-inflammatory signals (45).

Macrophage-related cytokines are also linked to the development of psoriasis. The levels of macrophage inflammatory protein (MIP)-1α, MIP-1β, and monocyte chemoattractant protein-1 were considerably elevated in patients with psoriasis vulgaris and positively associated with psoriasis area and severity index score (46). While MIF levels were elevated in the blood, MIF-positive staining in the psoriatic epidermis was notably reduced. MIF mRNA level decreased simultaneously in the psoriatic lesions, supporting this discovery (47). Further investigation is required to understand the disparity in MIF levels between the psoriatic epidermis and the circulation. The -173 GC genotype and the 6C haplotype of MIF polymorphisms are linked to an increased risk of plaque psoriasis in the Mexican population (48). Patients with psoriasis showed significantly lower frequencies of genotypes -794*CATT 5/7 and 7/7, while the CATT*5/MIF-173*C haplotype was more common (49).

### 3.3 Treatments for psoriasis involving macrophages

**Figure 2:**
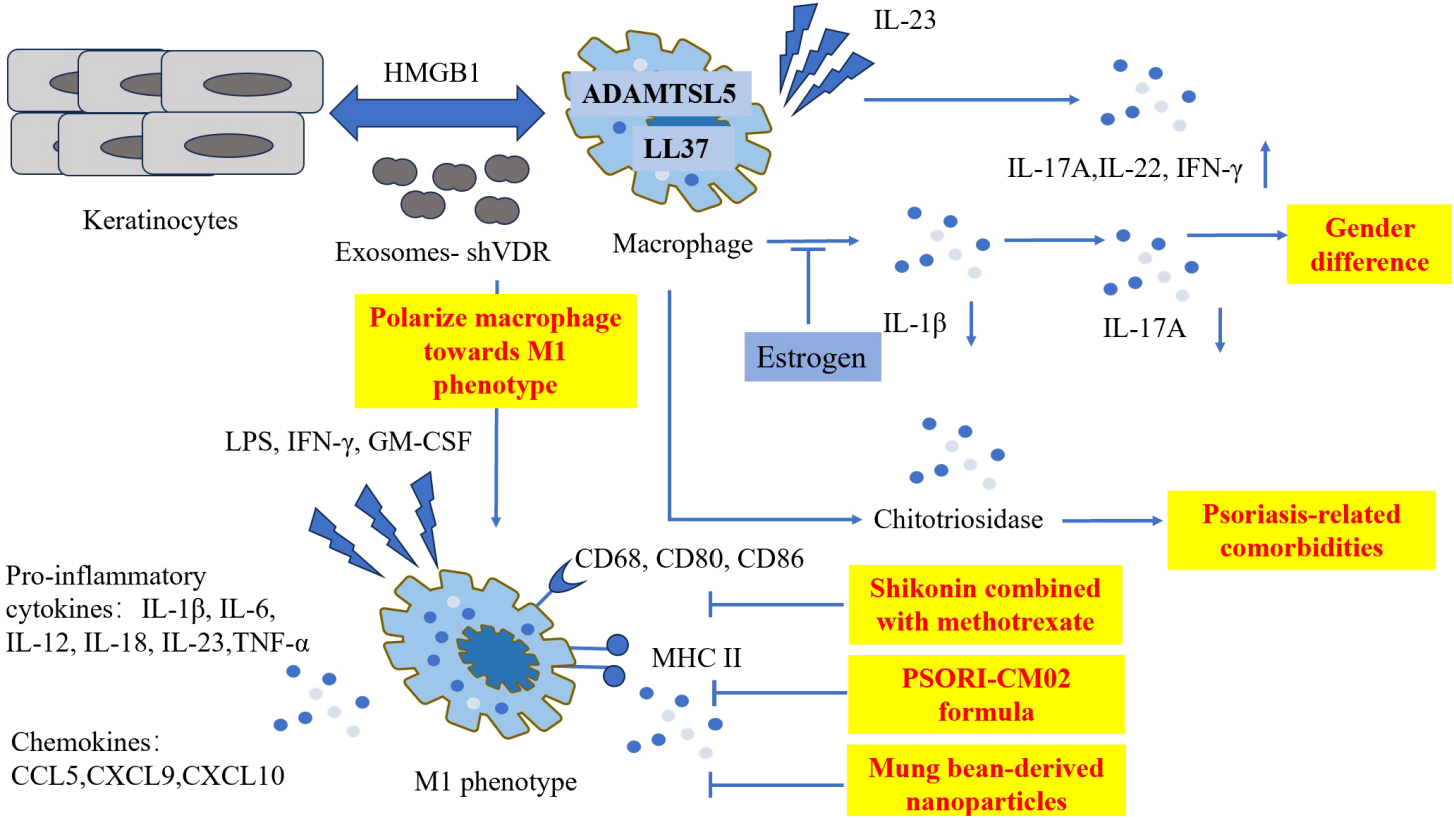
The pathogenetic roles of macrophages in psoriasis and treatments involving M1 phenotype. Macrophages co-express with two important psoriasis autoantigens LL-37 and ADAMTSL5. Macrophages triggered by IL-23 produce significant quantities of IL-7A, IL-22 and IFN-γ. Chitotriosidase secreted by activated macrophages is related to psoriasis-related comorbidities. Estrogen suppresses the production of IL-1β, furthermore reducing the level of IL-17A. Keratinocytes interact with macrophages via HMGB1, and exosomes derived from VDR-deficient keratinocytes polarize macrophages toward M1 phenotype, exaggerating the inflammation condition. Shikonin combined with methotrexate, PSORI-CM02 formula, and Mung bean-derived nanoparticles exhibit anti-psoriatic properties by hindering M1 polarization. ADAMTSL5, ADAMTS-Like Protein 5; CD, cluster of differentiation; CCL, C-C Motif Chemokine Ligand; CXCL, C-X-C Motif Chemokine Ligand; GM-CSF, granulocyte-macrophage colony-stimulating factor; HMGB1, High Mobility Group Box-1; IFN, interferon; IL, interleukin; LL-37, Cathelicidin; LPS, lipopolysaccharide; MHC, major histocompatibility complex; TNF, tumor necrosis factor; VDR, vitamin D receptor.

Shikonin is an organic matter extracted from the roots of Lithospermum erythrorhizon. Combining Shikonin with methotrexate has been demonstrated to hinder the advancement of psoriasis by controlling the polarization of macrophages. Administration of Shikonin and methotrexate in an imiquimod (IMQ)-induced psoriasis mice model can reduce the expression of F4/80 positive cells and decrease the mRNA levels of M1 macrophage markers (50). The PSORI-CM02 formula, a novel Chinese medicine, has been proven to have an anti-psoriatic effect. It can decrease macrophage infiltration, diminish M1 but increase M2 markers in IMQ-induced psoriasis mice (51). Etanercept, the first anti-TNF inhibitor, blocks the JAK/STAT3 pathway, decreasing the ratio of Th17/Treg and promoting M2 polarization, ultimately relieving psoriasis in mice (52). Application of Mung bean-derived nanoparticles topically can facilitate maintaining the balance of polarized macrophages and inhibit the activation of the NF-κB signaling pathway, leading to a reduction in skin inflammation (53). The pathogenetic roles of the macrophages in psoriasis and treatments involving the M1 phenotype are summarized in **Figure 2**.

## 4 Systemic sclerosis

Systemic sclerosis (SSc) is a paradigmatic rheumatic disease characterized by immune dysfunction-driven inflammation affecting multiple organs, finally leads to fibrosis. Skin involvement is among the most prominent manifestations of SSc. Raynaud’s phenomenon is the most prevalent skin lesion observed in SSc patients. Other skin lesions of SSc encompass puffy fingers, skin thickening and induration, digital ulcers, and hyperpigmentation. The exact cause of SSc is not well understood yet.

### 4.1 The characteristic of macrophage in SSc

In the skin of patients with SSc, there is a notable increase in the quantity of CD163+ cells located among collagen fibers when compared to the skin of healthy individuals (54). Macrophage signatures were found to be upregulated in early SSc patients compared to healthy controls. M2 and M1 macrophage signatures were present in 96% and 94% of patients, respectively. Furthermore, M2 and M1 signatures were associated with a higher extent of skin involvement, but also skin thickness progression rate prior to biopsy, an independent predictor of mortality (55). Dual phenotypic macrophages were recently identified in SSc disease. SSc patients exhibited elevated proportions of peripheral cells displaying M1, M2, and a combination of M1/M2 phenotypes in comparison to the control group (56). The transcriptome profile of macrophages in SSc shows increased activity in glycolysis, hypoxia, and mTOR signaling, while exhibiting decreased activity in IFN-γ response pathways (57). Single-cell transcriptome data have revealed three specific myeloid cell clusters in diffuse cutaneous SSc, including one macrophage cluster. This cluster expresses Fcγ receptor IIIA at high level, indicating a transition from normal CCR1+ and MARCO+ macrophages (58).

### 4.2 The pathogenic roles of the macrophages in SSc

Macrophages in SSc exhibit a profibrotic activation profile, meanwhile emit signaling molecules and have surface indicators linked to both M1 and M2 macrophage activation (59). M1 macrophage is associated with the beginning of fibrosis and accelerates its advancement in SSc. Research has shown that LPS-induced M1 macrophage pyroptosis contributes to fibrosis in SSc via the Cathepsin B/NLRP3/GSDMD pathway (60). In addition, ferroptosis presents in the bleomycin (BLM)-induced SSc mice model, where the M1 macrophage upregulates the expression of the ferroptosis driver Acyl-CoA synthetase long chain family member 4 and enhances its susceptibility to ferroptosis (61). Besides M1 macrophage, periostin contributes to the inflammation and fibrosis of SSc by potentially influencing M2 macrophages. Periostin-stimulated macrophages from healthy controls showed a substantial decrease in the proportion of M2 macrophages compared to those from SSc patients. Periostin stimulation led to a considerable upregulation of pro-fibrotic cytokines, chemokines, and extracellular matrix proteins in macrophages at the mRNA level (62).

Macrophages and fibroblasts contribute to the development of SSc by reciprocally activating each other. Macrophages show enhanced secretion of proinflammatory cytokines when stimulated with exosomes generated from fibroblasts of SSc patients. Collagen and fibronectin synthesis is greatly activated in fibroblasts when receiving signals from SSc exosome-stimulated macrophages (63). Co-culture investigations in Transwell experiments also demonstrated that SSc macrophages induce fibroblast activation (59). A self-assembled skin equivalent system was created to investigate the communication between macrophages and fibroblasts in SSc. The outcome provides more evidence supporting the mutual activation that relies partially on TGF-β (64). Depleting B cells has been suggested as a novel strategy for treating SSc, given that B cells can inhibit the differentiation of profibrotic macrophages. The extent of profibrotic macrophage activation induced by B cells is correlated with the fibrosis severity (65).

SSc-interstitial lung disease (ILD) is a complication associated with high morbidity and mortality. Immunohistochemistry analysis showed an accumulation of CD68+ and mannose-R+ macrophages in the lungs of SSc patients. Furthermore, single-cell RNA sequencing investigation of tissue-resident CD14+ pulmonary macrophages in SSc-ILD patients has shown an active profibrotic signature and increased Fibronectin 1 expression (66). Elevated levels of mixed M1/M2 macrophages in the circulation are linked to SSc-ILD, systolic pulmonary artery pressure, and the presence of anti-topoisomerase antibodies, which are established predictors of lung involvement in SSc (67). The upregulation of CCL18 and CD163 in the lungs of patients with SSc-ILD strongly implicates the pathogenetic roles of activated macrophages in this complication. Levels of CCL18 and CD163 are positively correlated with FibMax, an indicator for accessing lung fibrosis progression (68).

**Figure 3:**
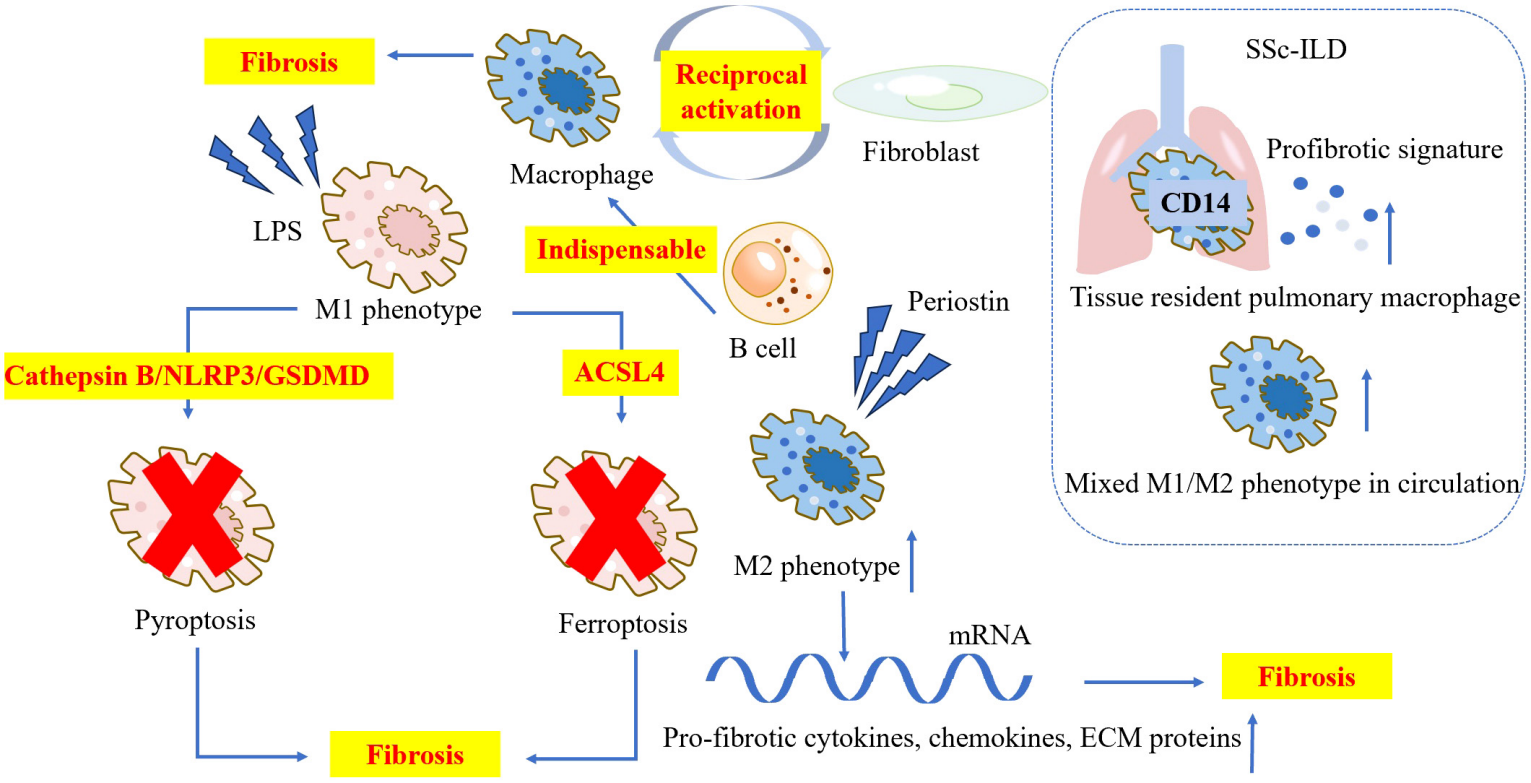
The characteristic and pathogenetic roles of the macrophages in SSc. Macrophages and fibroblasts mutually activate each other and contribute to the pathology in SSc. B cells promote the differentiation of profibrotic macrophages, and is indispensable for the progression of SSc. Periostin induces higher ratio of M2 macrophage and upregulates the mRNA level of pro-fibrotic cytokines, chemokines, and ECM proteins. M1 macrophage facilitates fibrosis by pyroptosis and ferroptosis. CD14+ tissue resident pulmonary macrophages in SSc-ILD patients’ lungs show an active profibrotic signature. Elevated levels of mixed M1/M2 phenotype macrophages are observed in the circulation of SSc-ILD patients. ACSL4, Acyl-CoA synthetase long chain family member4; CD, cluster of differentiation; ECM, extracellular matrix; GSDMD, Gasdermin D; LPS, lipopolysaccharide; NLRP3, NOD-like receptor thermal protein domain associated protein 3; SSc-ILD, systemic sclerosis-interstitial lung disease.

Levels of Serum MIF were considerably higher in both limited and diffuse SSc groups compared to healthy controls (69, 70). Microvascular endothelial cells and fibroblasts showed increased production of MIF when exposed to SSc serum, indicating the cellular source of MIF (70). MIF has the potential to serve as biomarkers and prognostic variables for pulmonary arterial hypertension (PAH) secondary to SSc. Patients with PAH related to SSc had elevated levels of MIF in their circulation compared to SSc patients without PAH. Patients with a higher New York Heart Association class exhibited higher levels of MIF (71). The MIF 7C haplotype is linked to an increased risk of SSc in the southern Mexican population and is correlated with increased MIF mRNA levels. MIF is associated with a proinflammatory response in SSc, as it correlates positively with the Th1 and Th17 cytokine profile (72). Except for MIF, Citrullinated vimentin, a biomarker of macrophage activation, was elevated in early diffuse-SSc compared to late diffuse-SSc (73). The characteristic and pathogenetic roles of the macrophages in SSc are summarized in **Figure 3**.

### 4.3 Treatments for SSc involving macrophages

Imatinib is a tyrosine kinase inhibitor typically used in the treatment of chronic myeloid leukemia. Notably, imatinib-loaded gold nanoparticles have demonstrated great efficacy in reducing IL-8 secretion, cell viability, and M2 polarization in alveolar macrophages (74). Nintedanib, another tyrosine kinase inhibitor, has shown promising antifibrotic effects in a SSc animal model. The underlying mechanism is associated with impaired M2 polarization of monocytes and reduced numbers of M2 macrophages (75). As for pulmonary fibrosis, an intractable problem in SSc patients, Zhang et al. proposed methyl-CpG-binding domain 2 (MBD2) as a novel therapeutic target. Depletion of MBD2 has been shown to prevent pulmonary fibrosis in a BLM-treated mouse model and to reduce the infiltration of M2 macrophage in the lungs of BLM-treated mice. MBD2 suppresses the SHIP expression and enhances PI3K/Akt signaling, thereby promoting the macrophage M2 phenotype (76). Ruxolitinib, a JAK inhibitor, exhibited anti-fibrosis properties in a BLM-SSc mouse model. *In vitro* experiments have revealed that ruxolitinib enhances macrophage efferocytosis when exposed to IFN, and reduced TGF-β- activated marker in fibroblasts derived from SSc-related pulmonary fibrosis tissues (77).

## 5 Systemic lupus erythematosus

Systemic lupus erythematosus (SLE) is a prototypical autoimmune disease characterized by complex pathophysiology and genetic susceptibility. The disease is defined by the involvement of multiple systems and organs, recurring flare-ups and remissions, and the emergence of various autoantibodies in the body. Untreated SLE can lead to permanent harm to organs and finally lead to death. Skin lesions are frequently observed in the majority of SLE patients. Nearly half of SLE presents with acute cutaneous lupus erythematosus, characterized by a butterfly-shaped rash over the cheeks and nose. Additionally, SLE patients may exhibit subacute and chronic cutaneous lupus erythematosus. Photosensitivity, alopecia, and oral mucosal ulcers are also frequently observed in SLE patients.

### 5.1 The pathogenic roles of the macrophages in SLE

Some scientists have suggested that M1 and M2 macrophages have distinct functions in the development of SLE. M1 macrophages exacerbate SLE, whereas M2 macrophages seem to alleviate its effects (78). The involvement of M2 macrophages in SLE is still a topic of debate. Other researchers observed a rise in the presence of CD163+ M2 macrophages in SLE skin and elevated soluble (s)CD163 levels in SLE patient blood specimens. Increased systemic and local CD163 expression indicates that M2 macrophages may contribute to the development of SLE as well (79). Furthermore, M2 macrophages have been suggested to play a role in the development of lupus nephritis. Urine sCD163 is highly associated with the current activity index of renal pathology and several particular pathological characteristics. M2 macrophages are a significant source of increased urine sCD163 levels, indicating its potential for predicting renal pathology (80).

Macrophages release ROS and inflammatory cytokines, which aggravate the inflammatory condition and tissue damage in SLE. Anti- dsDNA antibodies are crucial in the advancement of SLE. Anti-dsDNA antibodies can trigger NLRP3 inflammasome activation by binding to TLR-4 on macrophages, resulting in elevated mitochondrial ROS generation (81). Myeloid-derived suppressor cells may aggravate the IMQ-induced lupus model by enhancing TLR-7 pathway activation in macrophages. Mechanically, Myeloid-derived suppressor cells derived S100 Calcium Binding Protein A 8/9 increased IFN-γ secretion by macrophages, which then stimulated TLR-7 pathway activation in an autocrine manner (82). Activated lymphocyte-derived DNA induces macrophages to polarize toward M2b. M2b macrophages are distinguished by their production of inflammatory cytokines and their role in promoting inflammation condition, which is crucial in the progression of SLE (83). Activated lymphocyte-derived DNA-stimulated macrophages exhibit heightened glycolysis, reduced pentose phosphate pathway activity, and increased glycogenesis in glucose metabolism. The reduced pentose phosphate pathway activity ultimately resulted in increased levels of ROS (84).

Macrophages also play a role in the development of SLE by efferocytosis. Efferocytosis is the phagocytic elimination of apoptotic cells, and individuals with SLE show impairments in this process (85). The diminished efferocytosis is not an inherent defect but rather dependent on serum, linked to lower levels of C1q, C4, and C3 (86). Genes related to inflammation, autophagy, and signaling are upregulated in macrophages engulfing apoptotic cells from SLE patients (87). Efferocytosis capability differs between male and female mice. Female mice had a more pronounced impairment in macrophage efferocytosis compared to male mice, which could be reversed by administering male microbiota (85). SLE patients have been shown to exhibit elevated levels of urokinase-type plasminogen activator receptor expression. TLR-7 controls urokinase-type plasminogen activator receptor expression through ERK/c-JNK signaling and hinders macrophage efferocytosis (88). Tyro3 is a receptor that plays a role in identifying apoptotic cells in the process of efferocytosis. Autoantibodies targeting Tyro3 have been linked to increased disease activity in SLE and can hinder the ability of macrophage efferocytosis (89). Efferocytosis activity can be restored by co-culturing with human umbilical cord-derived mesenchymal stem cells. This reversal effect has been observed *in vitro* experiments and in SLE patients who underwent umbilical cord-derived mesenchymal stem cells transplantation (90). Bone marrow-derived mesenchymal stem cells release exosomes including miR-16 and miR-21, subsequently stimulate the anti-inflammatory transformation of macrophages. Furthermore, these macrophages exhibit enhanced efferocytosis ability and can be used to alleviate lupus nephritis (91).

### 5.2 Treatments for SLE involving macrophages

Azithromycin, a macrolide antibiotic, has emerged as a novel medication for SLE. *In vitro* experiments using macrophages that mimic the SLE phenotype have shown a reduction in M1 markers and an increase in M2 markers after azithromycin application, and this effect is dependent on Akt phosphorylation (92). Diffuse alveolar hemorrhage (DAH) is a potentially fatal complication of SLE. Serp-1, a rabbit myxomavirus-encoded serpin, has been shown to prevent the occurrence of SLE-associated DAH in a mouse model by modulating macrophage function. According to Zhuang et al., Serp-1 inhibits DAH by enhancing LXR-regulated M2 macrophage polarization and IL-10 production by KLH4 regulation (93). Additionally, PAM3, a TLR2/1 agonist, has shown promise in the treatment of SLE. It not only induces the differentiation of monocytes into an immunosuppressive M2 phenotype *in vitro* but also reduces disease severity in a lupus-prone mouse model (94).

## 6 Rosacea

Rosacea is a long-lasting inflammatory skin disorder identified by erythema and pustules. Macrophage infiltration is considered a frequently overlooked characteristic present in all kinds of rosacea (95). A large amount of CD68+ macrophages have been found to infiltrate the rosacea lesions (95, 96). Immune infiltration analysis also suggests that M1 macrophages play a significant role in rosacea (97).

### 6.1 The pathogenic roles of the macrophages in rosacea

Macrophages have been documented as participants in the deterioration mechanisms of rosacea. Guanylate Binding Protein 5 has been recognized as a crucial regulator of rosacea by promoting M1 macrophage polarization through the NF-κB signaling pathways (98). Elevated levels of the antimicrobial peptide LL-37 are commonly linked to the development of rosacea. LL-37 can enter macrophages’ cytoplasm via P2X7 receptor-mediated endocytosis and enhance NLRP3-mediated inflammasome activation in macrophages (99). ADAM-like metalloprotease Decysin-1 is considered to be associated with inflammation. Recent studies show that ADAM-like metalloprotease Decysin-1 may contribute to inflammation in rosacea by influencing the M1 polarization of macrophages (100).

### 6.2 Treatments for rosacea involving macrophages

Carvedilol, a nonselective beta-adrenoceptor antagonist, is an effective treatment for rosacea. *In vitro* studies have shown that carvedilol can reduce TLR-2 expression in macrophages, leading to decreased kallikrein related peptidase 5 secretion and LL-37 expression (101). Paeoniflorin, a monoterpenoid glycoside with various pharmacological activities, can alleviate rosacea-like inflammatory response by inducing suppressor of cytokine signaling 3 expression and suppressing the LPS-induced upregulation of TLR-2 and LL-37 via the ASK1-p38 cascade in macrophages (96). Artemisinin, the most effective antimalarial drug, decreases the presence of macrophages and immune cells in mice rosacea lesions, furthermore suppresses the production of chemokines associated with immune cells (102).

## 7 Bullous pemphigoid

Bullous pemphigoid (BP) is a deadly autoimmune dermatological disorder marked by initial red lesions and the subsequent formation of subepidermal blisters. The pathology of BP is linked to autoantibodies that target two hemidesmosomal proteins: BP180 and BP230.

### 7.1 The pathogenic roles of the macrophages in BP

There is a significant occurrence of CD163+ tissue-associated macrophages in BP. The increased levels of sCD163 in the serum of patients with BP compared to healthy individuals confirmed the activation of CD163+ tissue-associated macrophages. Chen et al. demonstrated that mice with macrophage deficiency were resistant to blister formation induced by pathogenic antibodies. In contrast, mice lacking T cells or B cells did not exhibit this resistance, indicating that macrophages, rather than T and B lymphocytes, play a pivotal role in the development of subepidermal blisters in experimental BP. Macrophages can facilitate the infiltration of neutrophils, a key step of experimental BP formation, and this mechanism relies on the activation or degranulation of mast cells (103). BP M2 macrophages showed a notable increase in both mRNA expression and production of CCL18 when exposed to IL-4 or IL-13 (104). Nuclear receptor related 1 belongs to the orphan nuclear receptor family and can regulate inflammation in both directions. Nuclear receptor related 1 is highly expressed in a specific group of cutaneous macrophages in patients with BP. This particular subgroup of macrophages in skin lesions is distinguished by elevated TNF levels and reduced expression of the anti-inflammatory marker CD163L1 (105).

### 7.2 Treatments for BP involving macrophages

Minocycline, a conventional medication for BP, has been shown to reduce the production of Th2 chemokines by M2 macrophages, thereby preventing the recruitment of Th2 cells and eosinophils to lesional skin in BP. While both CCL18 and CCL22 are Th2 chemokines implicated in BP, minocycline selectively suppresses the production of CCL18. The precise mechanism behind this selective effect remains to be elucidated (106). Dipeptidyl peptidase-4 inhibitors are associated with a higher incidence of BP. However, the concurrent use of lisinopril, a medication used to treat hypertension and heart failure, may counteract this risk. Lisinopril is capable of inhibiting the upregulation of matrix metalloproteinase and angiotensin-converting enzyme-2 in macrophages, thus exerting a mitigating effect on dipeptidyl peptidase-4 inhibitor-induced BP (107). T-cell immunoglobulin and mucin domain-3 is a well-recognized immune checkpoint molecule. Elevated levels of T-cell immunoglobulin and mucin domain 3 in macrophages within the affected skin of BP patients suggest its potential as a target for future immunotherapeutic interventions (108).

## 8 Melanoma

Melanomas are malignant tumors originating from melanocytes that can appear on any part of the body. Tumor-associated macrophages (TAMs) and other innate immune cells are crucial in chronic inflammatory processes that support tumor growth and advancement. M1 macrophages have immunostimulatory, anti-tumorigenic, and anti-angiogenic properties, while M2 macrophages support tumor growth and angiogenesis.

### 8.1 The characteristic of macrophage in melanoma

Studies have shown that invasive melanomas have a greater quantity of CD68+ and CD163+ TAMs in comparison to benign nevi (109). TAMs in melanoma are a diverse and constantly changing group, with a subset of unpolarized CD68+/CD163–/iNOS– macrophages consistently existing (110). Different stages of melanomas display distinct macrophage constituents. During the initial phase of malignant melanoma, the number of M1 intratumoral macrophages is lower than that of the M2 population. As the disease advanced, M1 macrophage recruitment was quickly and increasingly surpassed by an upsurge in M2 TAMs (111).Macrophages’ function differs based on their location. Stromal macrophages have a unique transcriptional profile compared to those found in tumor nests, as they are reprogrammed to take on DC activity (112). The quantity and composition of macrophages are associated with the outcome of melanoma. High numbers of CD68+ macrophages inside tumor cell nests are linked to recurrence, while a low proportion of CD163+ macrophages in the tumor stroma is related to recurrence and, in initial melanomas, also with poor overall survival (109). The state of macrophage polarization is linked to the level of lymphocytic infiltration in melanoma, which also impacts the prognosis (110).

### 8.2 The pathogenic roles of the macrophages in melanoma

Increasing evidence has revealed that macrophages are implicated in melanoma migration. CD163+ macrophages found within the tumors are associated with the development of metastases (113). Angiogenesis is a crucial step in the preparation of lymph nodes for melanoma metastasis. Exosomes from melanoma cells stimulate the generation of granulocyte-macrophage colony stimulating factor in pre-metastatic lymph nodes. Granulocyte-macrophage colony stimulating factor could activate hypoxia-inducible factor (HIF)-1α in M1 macrophages and HIF-2α in M2 macrophages. HIF-1α stimulates new blood vessel formation, whereas HIF-2α contributes to the structural normalization of newly formed blood vessels (114). TAMs promoted endothelial cell movement, tube creation, and tumor development through TAM-derived adrenomedullin. Adrenomedullin possess endocrine and paracrine activities simultaneously. The paracrine effect is mediated by the endothelial NOS signaling pathway, while the autocrine effect induces macrophages to polarize toward the M2 phenotype (115).

Tumor cells and TAMs interactions are crucial for initiating tumor cell motility. TAMs can transmit cytoplasmic modules to tumor cells, enhancing tumor cell motility and dissemination (116). Another hypothesis for metastasis mentions the fusion of macrophages with tumor cells (MTFs). After being injected subcutaneously into nude mice, cultivated MTFs spread and formed metastatic tumors at remote locations. The cultivated MTFs consistently displayed pan-macrophage markers, M2 polarization markers, and melanocyte-specific markers (117). HMGB1 has a significant role in the growth and spread of murine melanoma. HMGB1 is secreted by melanoma tumor cells as a consequence of hypoxia, and could increase M2-like TAMs accumulation and an create an IL-10-rich TME (118). CD34- melanoma-initiating cells rely on M2 macrophages for their survival and growth. This discovery provides additional confirmation that macrophages play a role in the distant spread of melanoma (119).

### 8.3 Treatments for melanoma involving macrophages

Transitioning the polarization state of TAMs from the tumor-favoring M2 phenotype to the anti-tumor M1 phenotype is a promising strategy in oncotherapy. Chemotherapy occupies an important component position in combination treatments of melanoma. Doxorubicin-loaded polysaccharide hydrogels have demonstrated effective polarization of TAMs toward the M1 phenotype (120).

In addition to traditional chemotherapy drugs, researchers are now exploring new methods by regulating macrophage polarization to treat melanoma. TLR-7/8 agonists, such as resiquimod (RES) and telratolimod, can induce the polarization of macrophages toward the M1 phenotype. Bexarotene (BEX), a highly affinity selective retinoid X receptor, can reduce M2 polarization. A dual macrophage polarizer was created by mixing BEX with RES to enhance the M1 phenotype while inhibit the M2 phenotype. This combination exhibited incomparable inhibitory effects on B16F10 cells (121). Tumor-associated adipocyte exhibits a transformed pro-tumorigenic characteristic which can attract monocytes and stimulate their transformation into the M2 phenotype. Telratolimod is encapsulated within the lipid droplets of adipocytes and is intended to be discharged at the tumor site. Injecting drug-loaded adipocytes boosted tumor-inhibiting M1 macrophages in primary and distant tumors, halting tumor growth in a melanoma model (122). These innovative treatments have demonstrated anti-tumor effects in animal and cell models, but they have not yet been implemented in clinical practice.

## 9 Cutaneous T-cell lymphoma

Cutaneous T-cell lymphoma (CTCL) is a rare kind of lymphoma originating in the skin, and consists of a collection of subtypes with different clinical manifestations, histological features, and prognosis. Mycosis fungoides (MF) and Sézary syndrome (SS) are the two main types of CTCL (123). While CTCL may progress slowly in its initial stages, it can result in considerable morbidity and mortality as it proceeds (124).

### 9.1 The characteristic of macrophage in CTCL

A prominent subtype of M2 TAM expressing PD-1 has been found in CTCL TME, and playing an immunosuppressive role. Lenalidomide is an immunomodulatory drug typically used in treating hematological malignancies. Anti–PD-L1 combined with lenalidomide induces functional changes in TAMs, thereby enhancing phagocytic activity and impairing migration of M2-like TAMs and augmenting T cell proliferation to improve antitumor immunity. Combining anti-PD-L1 and lenalidomide treatment induces a functional transition from a PD-1+ M2 phenotype toward a proinflammatory M1 phenotype *in vitro*. Meanwhile, this transformation enhances phagocytic activity by blocking NF-κB and JAK/STAT (125).

The polarization state of macrophages in CTCL TME is not static. Granulomatous MF shows a transition of macrophage polarization from M1 in the initial phases to M2 in the later stages (126). The quantity of macrophages varies depending on the tumor stage, with a notably greater amount of CD68+ macrophages in the tumor-stage compared to early-stage folliculotropic MF (127).

Granulomatous slack skin is a very uncommon type of CTCL distinguished by a high quantity of macrophages. Macrophages in granulomatous slack skin are divided into three distinct subpopulations with unique transcript characteristics (128):

- The CD163+/CD206+ cluster displays a TAM M2-like phenotype and expresses markers involved in T-cell interaction and tumor progression.
- The apolipoprotein C1+/APOE+ cluster has a non-M1 or -M2 phenotype and may be associated with lipid metabolism.
- The CD11c+/lysozyme+ cluster demonstrates an M1-like phenotype and expresses matrix metalloproteinase-9 strongly.

### 9.2 The pathogenic roles of the macrophages in CTCL

The interaction between malignant T cells and macrophages is extensively studied in CTCL TME. A subtyping system has been created using the genetic characteristics of malignant T cells and the surrounding TME that promotes tumor growth (129). The interaction between malignant CTCL cells and CCL13+ macrophages has been demonstrated to promote tumor growth by increasing S100 Calcium Binding Protein A9 levels and activating NF-κB (130). Similar intercellular communications have been observed in the transformed CTCL tumor ecosystem. Malignant T cells that express MIF interact with macrophages, and B cells that express CD74 are also involved in this interaction (131).

Macrophage enrichment has a role in creating an immunosuppressive TME. Elimination of M2-like TAMs using liposomes containing clodronate (the first-generation bisphosphonate treating osteoporosis) has been demonstrated to postpone the progression of CTCL (132). Furthermore, the expressions of vascular markers also decrease by macrophage exhaustion, suggesting macrophages are implicated in both the advancement of CTCL and neoangiogenesis. CCR2 inhibitor, which hinders the movement of monocytes through CCR2, can lead to the reduction of macrophages. Mice treated with CCR2 inhibitor showed significantly reduced tumor sizes and weights compared to the control group, providing more evidence of the adverse impact of macrophages in CTCL (133).

Macrophages play a predictive role in the progression of CTCL, with the quantities of CD163+ cells in affected skin and serum sCD163 levels correlating with disease advancement (134). Another study suggests that the CD163/CD68 ratio should be used to evaluate TAMs instead of focusing on the total TAM count. A high ratio of CD163/CD68 in tumor stage MF and SS suggests M2 polarization of TAMs, which is associated with tumor advancement. Serum levels of sCD163 and CCL22 can indicate M2 load and may serve as indicators for evaluating disease progression (135). However, there is still no consensus on the relationship between CD163+ cells with tumor progression. Some researchers suggest that the proportion of CD206+ cells, as opposed to CD163+ cells, increases in correlation with tumor advancement (136).

### 9.3 Treatments for CTCL involving macrophages

BEX has been authorized for the treatment of relapsed CTCL after at least one prior systemic therapy. BEX’s clinical benefits are partly attributable to its ability to decrease the synthesis of CCL22 by M2 TAMs (137). IFNs are efficacious in treating advanced-stage MF, potentially by influencing M2 TAMs as well. Mechanistically, IFN-α2a and IFN-γ reduce CCL17 and CCL18 expression and synthesis, while raising CXCL10 and CXCL11 levels in M2 macrophages (138).

## 10 Conclusion and prospect

Numerous immune cells get involved in the pathogenesis of inflammatory skin diseases and skin tumors. In this review, we aim to understand the pathogenesis from the perspective of macrophages. Due to their complex functions and dynamic polarization states, macrophages are extensively implicated in the occurrence of AD, psoriasis, SSc, SLE, rosacea, BP, melanoma and CTCL. The mechanism of macrophages in these conditions is multifaceted, including intercellular interactions (macrophages and B cells, T cells, keratinocytes, basophils and fibroblasts), cell death (ferroptosis and pyroptosis), and cell functions (autophagy and efferocytosis). Additionally, multiple signaling pathways and molecules, such as exosomes, ILs, CCLs, CXCLs, are also involved.

In the future, we anticipate that more macrophage-related indicators can be developed to assess the disease severity, prognosis and complication occurrence and to guide more precise treatment. Furthermore, targeting the number and polarization state of the macrophages holds promise for the exploration of new therapeutic approaches. For example, M2 macrophages are considered to play immunosuppressive roles in the TME. Research may focus on depleting M2 macrophages or converting them to an anti-tumoral M1 phenotype within the TME with safe medications. The investigation of macrophages in inflammatory skin diseases and skin tumors remains a vibrant research area and we are confident that patients will benefit from these advancements in the future.
